# Relationship of Circulating Soluble Urokinase Plasminogen Activator Receptor (suPAR) Levels to Disease Control in Asthma and Asthmatic Pregnancy

**DOI:** 10.1371/journal.pone.0060697

**Published:** 2013-04-02

**Authors:** István Ivancsó, Gergely Toldi, Anikó Bohács, Noémi Eszes, Veronika Müller, János Rigó, Barna Vásárhelyi, György Losonczy, Lilla Tamási

**Affiliations:** 1 Department of Pulmonology, Semmelweis University, Budapest, Hungary; 2 1st Department of Pediatrics, Semmelweis University, Budapest, Hungary; 3 1st Department of Obstetrics and Gynecology, Semmelweis University, Budapest, Hungary; 4 Department of Laboratory Medicine, Semmelweis University, Budapest, Hungary; The University of Kansas Medical Center, United States of America

## Abstract

Asthma has a high burden of morbidity if not controlled and may frequently complicate pregnancy, posing a risk for pregnancy outcomes. Elevated plasma level of the inflammatory biomarker soluble urokinase plasminogen activator receptor (suPAR) is related to a worse prognosis in many conditions such as infectious, autoimmune, or pregnancy-related diseases; however the value of suPAR in asthma and asthmatic pregnancy is unknown. The present study aimed to investigate the suPAR, CRP and IL-6 levels in asthma (asthmatic non-pregnant, ANP; N = 38; female N = 27) and asthmatic pregnancy (AP; N = 15), compared to healthy non-pregnant controls (HNP; N = 29; female N = 19) and to healthy pregnant women (HP; N = 58). The relationship between suPAR levels and asthma control was also evaluated. The diagnostic efficacy of suPAR in asthma control was analyzed using ROC analysis. IL-6 and CRP levels were comparable in all study groups. Circulating suPAR levels were lower in HP and AP than in HNP and ANP subjects, respectively (2.01 [1.81–2.38] and 2.39 [2.07–2.69] vs. 2.60 [1.82–3.49] and 2.84 [2.33–3.72] ng/mL, respectively, p = 0.0001). suPAR and airway resistance correlated in ANP (r = 0.47, p = 0.004). ROC analysis of suPAR values in ANP patients with PEF above and below 80% yielded an AUC of 0.75 (95% CI: 0.57–0.92, p = 0.023) and with ACT total score above and below 20 an AUC of 0.80 (95% CI: 0.64–0.95, p = 0.006). The cut-off value of suPAR to discriminate between controlled and not controlled AP and ANP was 4.04 ng/mL. In conclusion, suPAR may help the objective assessment of asthma control, since it correlates with airway resistance and has good sensitivity in the detection of impaired asthma control. Decrease in circulating suPAR levels detected both in healthy and asthmatic pregnant women presumably represents pregnancy induced immune tolerance.

## Introduction

Asthma is a chronic inflammatory disease of the airways characterized by variable and recurring symptoms, local inflammation, reversible airflow obstruction, and bronchospasm (Global Initiative for Asthma (GINA) guideline; [1]). Asthma is a prevalent chronic disease which is not optimally controlled in up to 50% of cases worldwide. It has a high burden of morbidity especially if not controlled [1]; however blood biomarkers to identify patients at risk are not available. Systemic inflammation in asthma causes an acute phase response, as it was shown by increased level of C-reactive protein (CRP) related to total immunglobulin (Ig)E levels or respiratory symptoms of asthma [2], [3]. Circulating interleukin (IL)-6 is also a marker of inflammation [4] which is elevated in asthmatic patients compared to healthy individuals and which further increases after allergen challenge [5], [6]. However, these markers are not sufficiently sensitive to asthma control.

Asthma is one of the most common chronic diseases complicating pregnancy [7]. It represents a risk for maternal and fetal morbidities, including preterm delivery, gestational hypertension, preeclampsia, low birth weight, and neonatal mortality [8], [9]. On the other hand, pregnancy may also influence the control of asthma, leading to the deterioration of symptoms in one-third of pregnant women [10]. Asthmatic pregnancy is characterized with alteration in immune phenotype [11] and cytokine patterns [12]. Due to clinical and immunological interactions between asthma and pregnancy women with asthma during pregnancy represent a special challenge for asthma specialists. Optimal asthma control during pregnancy is associated with lower risk of maternal and neonatal complications [13]; therefore the identification of pregnant women at risk of not controlled asthma and sufficient symptom control is important in this patient group. However, although some clinical factors including the severity of asthma before pregnancy, disease worsening in previous pregnancies and impaired asthma-specific quality of life in early pregnancy are predictive for the loss of asthma control during pregnancy [9], [10], [14], [15], to date systemic markers related to asthma control determinants or lung function in asthmatic pregnancy are missing.

Over the recent years, soluble urokinase plasminogen activator receptor (suPAR) has been emerged as a valuable indicator of the activation state of the immune system. Urokinase-type plasminogen activator receptor (uPAR) is expressed on various cell types, including immune, smooth muscle and endothelial cells [16], [17]. This membrane protein may be cleaved from the cell surface, thus forming a free soluble receptor, suPAR [18]. suPAR is detectable in low, but constant concentrations in plasma of healthy individuals [18], [19]. In contrast to C-reactive protein (CRP), the suPAR levels are not affected by diurnal variation and fasting state [20]. suPAR concentrations are resistant even to freezing and thawing of plasma samples [21]. suPAR’s high stability in plasma samples makes it an ideal candidate as a potential clinical biomarker for inflammation. In many conditions such as in infectious [22], [23], [24], [25], autoimmune [26], [27], and neoplastic diseases [28], pregnancy [29] and pregnancy-related disorders [30], higher than normal levels of suPAR were measured. According to the data available, inflammatory response leads to elevated plasma suPAR levels in many inflammatory diseases [31] which is predictive to a worse prognosis [20], [22], [23], [25], [28], [32]. Furthermore, suPAR appears to be a pre-clinical biomarker of preeclampsia in late pregnancy [30].

In the present study we aimed to investigate the suPAR levels in asthma and asthmatic pregnancy. The possible relationship between suPAR and asthma control determinants was also evaluated. In order to have a more complete view on systemic immune activation, circulating CRP and IL-6 levels were also evaluated.

## Methods

### Ethics Statement

Written informed consent was obtained from all subjects, and our study was reviewed and approved by an independent ethical committee of the institution (Institutional and Regional Research Ethics Committee of Semmelweis Medical University). Laboratory studies and interpretations were performed on coded samples lacking personal and diagnostic identifiers. The study was adhered to the tenets of the most recent revision of the Declaration of Helsinki.

### Study participants

The study had a cross-sectional design. 29 healthy non-pregnant (HNP) controls (19 females and 10 males), 58 healthy pregnant (HP) women, 38 asthmatic non-pregnant (ANP) patients (27 females and 11 males) and 15 asthmatic pregnant (AP) women were enrolled. Asthmatic patients were assessed at their regular visit at the outpatient clinic of the Department of Pulmonology, Semmelweis University. They had persistent disease and asthma had been diagnosed according to the current guidelines [1] at least 6 months prior to the study. Exclusion criteria were hypertension, diabetes mellitus, autoimmune disease, angiopathy, renal disorder, maternal or fetal infection, fetal congenital anomaly, multi-fetal gestation, current smoking or more than 5 pack years of smoking history, any other chronic disease (except for allergic rhinitis), and acute infection within four weeks of measurement. Patients were asked not to use their medication 12 hours before visits. Healthy non-pregnant controls were volunteer blood donors and had a negative history and negative status upon detailed physical and routine laboratory examination. Healthy pregnant subjects were recruited when attending their scheduled visit at the 1^st^ Department of Obstetrics and Gynaecology, Semmelweis University.

### Laboratory procedures

Plasma was isolated from EDTA anticoagulated fasting blood samples and stored at –80°C until measurement. Plasma suPAR concentrations were measured with the suPARnostic Flex ELISA assay (ViroGates A/S, Birkerød, Denmark). High-sensitivity (hs-)CRP levels were measured using commercially available tests (Roche Diagnostics GmbH, Mannheim, Germany). For the determination of erythrocyte sedimentation rate (ESR), the Westergren method was performed according to ICSH specifications (International Council for Standardization in Haematology, 1993) on undiluted EDTA anticoagulated blood samples using glass pipettes (Greiner Bio-One, Kremsmuenster, Austria). During sedimentation, the pipettes were mounted vertically on appropriate supporting racks and kept at room temperature, which never exceeded 25°C.

### Lung function measurement and asthma control evaluation

Lung function was measured by means of electronic spirometer (PDD-301/s, Piston, Budapest, Hungary) according to the American Thoracic Society (ATS) guidelines [33]. Three technically acceptable maneuvers were performed and the highest was used. Forced expiratory volume in one second (FEV_1_), peak expiratory flow rate (PEF), and airway resistance (Raw) were measured. Asthma control was assessed using the Asthma Control Test (ACT) suggested by the current [**1**] guideline.

### Statistics

Data are expressed as median [interquartile range]. CRP values below the level of detection (1 mg/L) were regarded as 1 mg/L. IL-6 values below the level of detection (1.5 pg/mL) were regarded as 1.5 pg/mL. Comparisons between the study groups were made with Kruskal-Wallis tests. Correlation analyses were performed using Spearman’s tests due to non-normal distribution of data. Area Under Curve (AUC) values of Receiver Operating Characteristics (ROC) curves were calculated using standard methods and data are presented as AUC ROC (95% CI). p values < 0.05 were considered significant. Statistics were calculated using the SPSS software (version 20.0, SPSS, Inc. Chicago, IL, USA).

## Results

### Clinical characteristics

Clinical data and inflammatory parameters are summarized in Table 1. The median age of participants was higher in the ANP group compared to the HP and AP groups (39 [32–58] vs. 31 [28]–[35] and 29.5 [26]–[32] years, respectively, p = 0.0001). Sampling was performed in the second or third trimester of gestation in all pregnant women, however, gestational age at blood collection was lower in the AP than in the HP group (27.5 [24–33.5] vs. 36 [34]–[38] weeks, p  =  0.0002). Gestational age at delivery and fetal birth weight were comparable in the pregnant groups. No difference was detected either in parameters describing the severity or control of asthma or in daily dose of inhaled corticosteroids between the ANP and AP groups (Table 1).

**Table 1:** Clinical data and inflammatory parameters of the four study groups (median [interquartile range]).

	HNP(n = 29)	HP(n = 58)	ANP(n = 38)	AP(n = 15)
Female / male	19 / 10	–	27 / 11	
Age (years)	36 [30–52]	31 [28]–[35]	39 [32–58]^b^	29.5 [26]–[32] ^c^
Gestational age at sampling (weeks)	–	36 [34]–[38]	–	27.5 [24–33.5]^b^
Gestational age at delivery (weeks)	–	39 [38]–[40]	–	38 [38]–[39]
Fetal birth weight (grams)	–	3255 [3090–3745]	–	3320 [3000–4000]
FEV_1_ (% of predicted)	–	–	89.0 [83.5–98.0]	96.0 [82.0–108.0]
PEF (% of predicted)	–	–	90.5 [77.5–100.0]	89.0 [75.0–107.0]
R_aw_ (% of predicted)	–	–	150 [92–179]	105 [84–127]
ACT total score	–	–	21.5 [17.5–24.0]	22.0 [18.5–25.0]
Daily dose of ICS (beclomethasone equivalent, μg)	–	–	1000 [800–1000]	1000 [375-1000]
CRP (mg/L)	2.50 [1.00–3.90]	3.85 [2.08–6.53]	3.00 [3.00–5.00]	7.00 [3.00–9.00]^a^
suPAR (ng/mL)	2.60 [1.82–3.49]	2.01 [1.81–2.38]^a^	2.84 [2.33–3.72]^b^	2.39 [2.07–2.69]^c^
IL-6 (pg/mL)	1.50 [1.50–1.70]	1.50 [1.50–2.18]	1.64 [1.50–3.36]	1.82 [1.50–3.43]

HNP – healthy non-pregnant; HP – healthy pregnant; ANP – asthmatic non-pregnant; AP – asthmatic pregnant; FEV_1_ – forced expiratory volume in 1 second; PEF – peak expiratory flow rate; R_aw_ – airway resistance; ACT – asthma control test; ICS – inhaled corticosteroid; CRP – C-reactive protein; suPAR – soluble urokinase plasminogen activator receptor; IL-6 – interleukin 6; ^a^ p < 0.05 vs. HNP, ^b^ p < 0.05 vs. HP, ^c^ p < 0.05 vs. ANP.

### Comparison of circulating marker levels among the four groups

**Figure 1:**
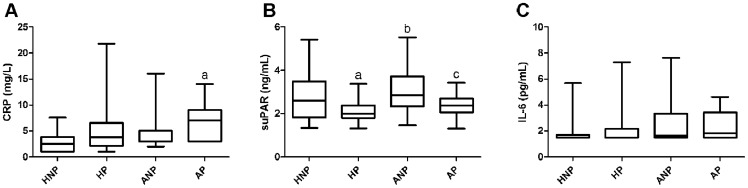
Circulating CRP (A), suPAR (B) and IL-6 (C) levels measured in healthy non-pregnant and pregnant, and asthmatic non-pregnant and pregnant subjects. HNP – healthy non-pregnant; HP – healthy pregnant; ANP – asthmatic non-pregnant; AP – asthmatic pregnant; CRP – C-reactive protein; suPAR – soluble urokinase plasminogen activator receptor; IL-6 – interleukin 6; ^a^p<0.05 vs. HNP, ^b^p<0.05 vs. HP and ^c^p<0.05 vs. ANP.

Treated asthma itself did not alter suPAR levels as ANP and HNP groups had similar circulating suPAR levels (p>0.05; Figure 1). Circulating suPAR values were lower in the HP and AP than in the HNP and ANP subjects, respectively (2.01 [1.81–2.38] and 2.39 [2.07–2.69] vs. 2.60 [1.82–3.49] and 2.84 [2.33–3.72] ng/mL, respectively, p = 0.0001). IL-6 levels were comparable in all study groups. CRP values were comparable in the HNP, ANP and HP groups and were higher in the AP group compared to the HNP (7.00 [3.00–9.00] vs. 2.50 [1.00–3.90] mg/L, p = 0.005) but not to the HP group (p>0.05) (Figure 1).

### Relationship of inflammatory markers to asthma control determinants and neonatal birth weight

**Figure 2:**
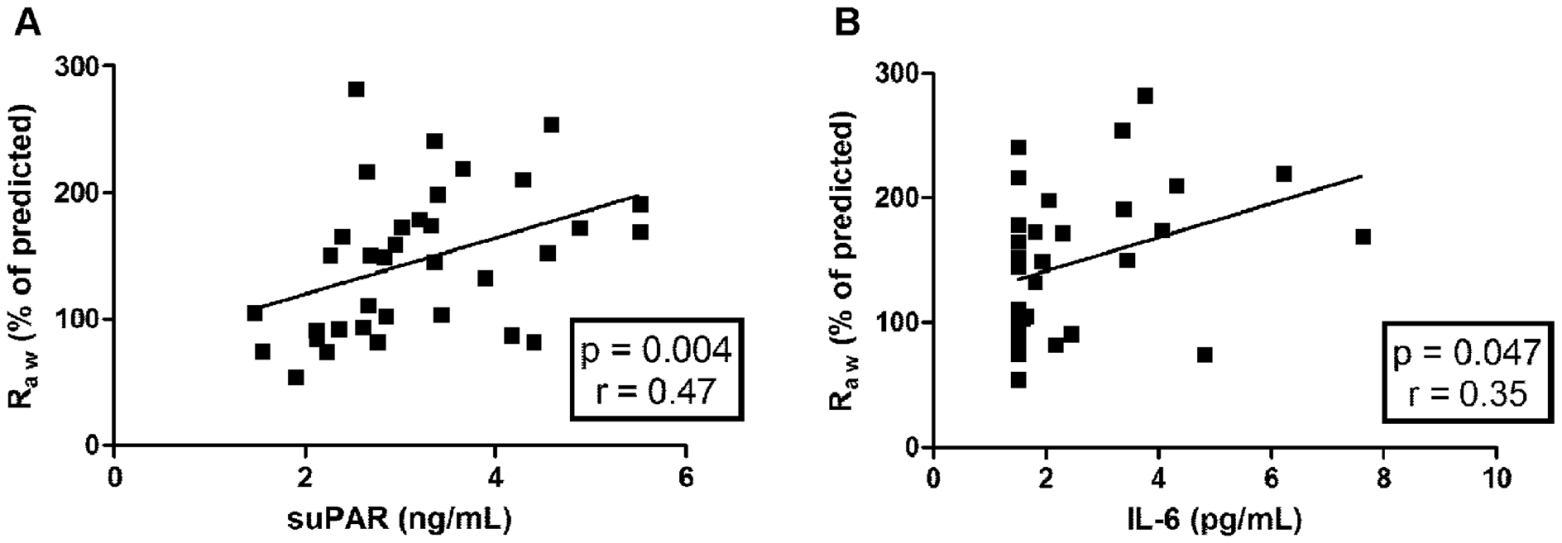
Positive correlation of airway resistance (R_aw_) to circulating suPAR (A) and to circulating IL-6 (B) levels in asthmatic non-pregnant patients. suPAR – soluble urokinase plasminogen activator receptor; IL-6 – interleukin 6.

PEF, R_aw_ and ACT values were correlated with suPAR, IL-6 and CRP levels, respectively, in the ANP and AP groups. Correlation analysis revealed a positive correlation between R_aw_ and suPAR levels (p  =  0.004, r  =  0.47) as well as R_aw_ and IL-6 levels (p  =  0.047, r  =  0.35) in the ANP group (Figure 2), however no such correlation was detected in the AP group. Similarly, no correlation was observed between clinical parameters of asthma and CRP in both asthmatic groups, and no correlation was detected between the investigated inflammatory markers and neonatal birth weight in the pregnant groups. Furthermore, circulating suPAR levels were not influenced by the daily inhaled corticosteroid dose of the patients.

**Figure 3:**
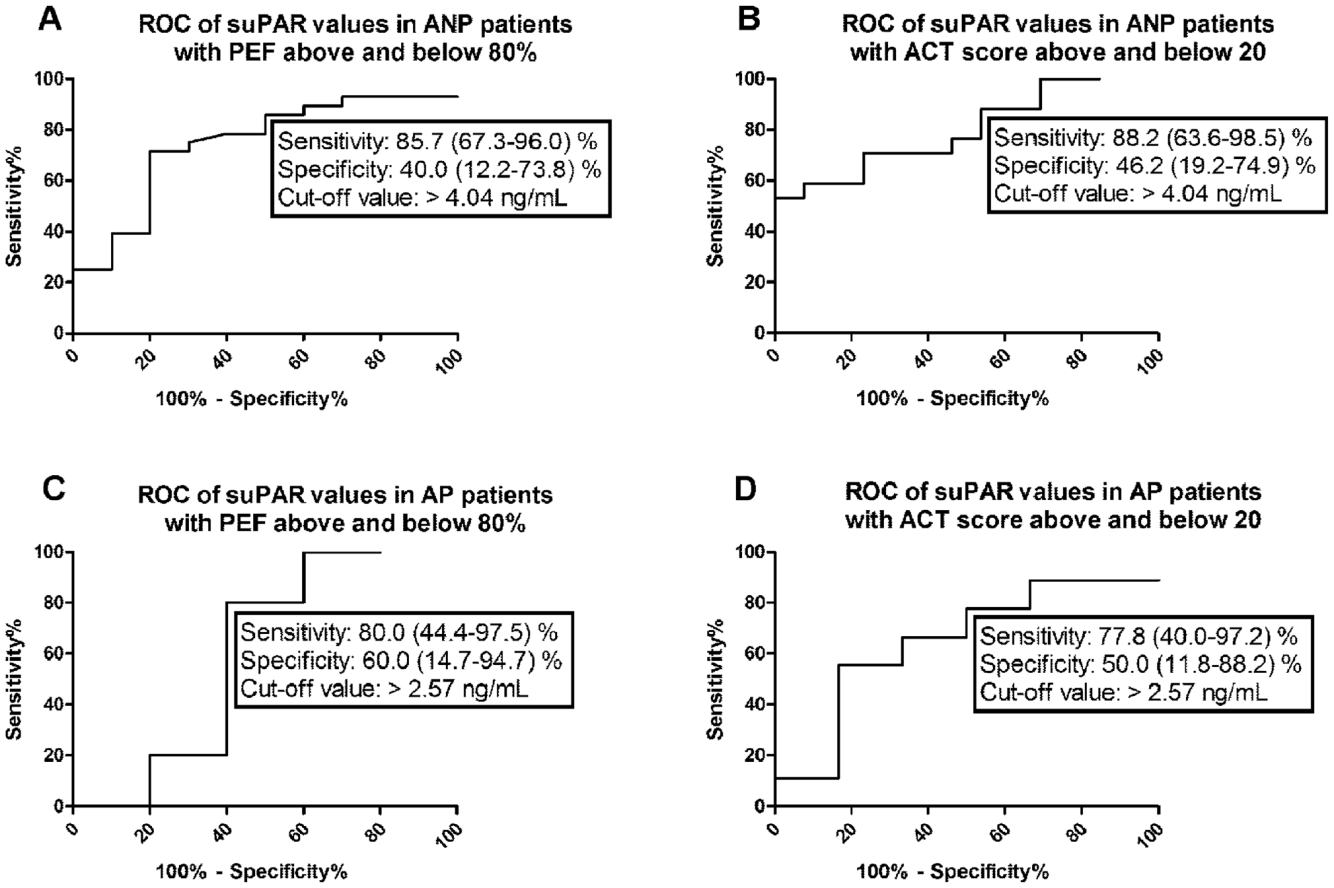
ROC analyses of suPAR values in asthmatic non-pregnant patients (A, B) and asthmatic pregnant patients (C, D) with good and impaired asthma control. ANP – asthmatic non-pregnant, AP – asthmatic pregnant; PEF – peak expiratory flow rate, ACT – Asthma Control Test total score.

As current asthma guideline suggests PEF > 80% and ACT total score > 20 as the main determinants of well-controlled asthma, ROC analyses of asthmatic patients’ data were performed in subgroups of AP and ANP with PEF above and below 80% and ACT total score above and below 20. ROC analyses in different subgroups of ANP and AP patients according to PEF and ACT scores proved to be statistically significant only in case of suPAR in the ANP group, while p values were higher than 0.05 for IL-6 and CRP and for all three biomarkers in the AP group. ROC analysis of suPAR values in ANP patients with PEF above and below 80% yielded an AUC of 0.75 (95% CI: 0.57–0.92, p = 0.023, Figure 3). ROC analysis of suPAR values in ANP patients with ACT score above and below 20 yielded an AUC of 0.80 (95% CI: 0.64–0.95, p = 0.006, Figure 3). The cut-off value of suPAR to discriminate between ANP patients with PEF above and below 80% was 4.04 ng/mL (sensitivity% (95% CI): 85.7 (67.3–96.0), specificity% (95% CI): 40.0 (12.2–73.8), Table 2). The cut-off value of suPAR to discriminate between ANP patients with an ACT score above and below 20 was 4.04 ng/mL (sensitivity% (95% CI): 88.2 (63.6–98.5), specificity% (95% CI): 46.2 (19.2–74.9); Table 2).

**Table 2:** Cut-off values of the inflammatory markers.

	Sensitivity	Specificity	Cut-off value
ANP according to PEF, CRP	74.1 (53.7–88.9) %	30.0 (6.7–65.3) %	> 4.50 mg/L
ANP according to PEF, suPAR	85.7 (67.3–96.0) %	40.0 (12.2–73.8) %	> 4.04 ng/mL*
ANP according to PEF, IL-6	78.3 (56.3–92.5) %	40.0 (12.2–73.8) %	> 2.90 pg/mL
ANP according to ACT, CRP	31.3 (11.0–58.7) %	76.9 (46.2–95.0) %	> 4.50 mg/L
ANP according to ACT, suPAR	88.2 (63.6–98.5) %	46.2 (19.2–74.9) %	> 4.04 ng/mL*
ANP according to ACT, IL-6	81.8 (59.7–94.8) %	45.5 (16.8–76.6) %	> 2.90 pg/mL
AP according to PEF, CRP	60.0 (26.2–87.8) %	66.7 (9.4–99.2) %	> 7.50 mg/L
AP according to PEF, suPAR	80.0 (44.4–97.5) %	60.0 (14.7–94.7) %	> 2.57 ng/mL
AP according to PEF, IL-6	70.0 (34.8–93.3) %	50.0 (6.8–93.2) %	> 2.48 pg/mL
AP according to ACT, CRP	62.5 (24.5–91.5) %	60.0 (14.7–94.7) %	> 7.50 mg/L
AP according to ACT, suPAR	77.8 (40.0–97.2) %	50.0 (11.8–88.2) %	> 2.57 ng/mL
AP according to ACT, IL-6	75.0 (34.9–96.8) %	50.0 (11.8–88.2) %	> 2.48 pg/mL

Table shows the cut-off values of the inflammatory markers to discriminate between patients with PEF above and below 80% and ACT total score above and below 20, respectively in the asthmatic non-pregnant (ANP) and asthmatic pregnant (AP) groups.

PEF – peak expiratory flow rate; CRP – C-reactive protein; suPAR – soluble urokinase plasminogen activator receptor; IL-6 – interleukin 6; ACT – asthma control test; * p < 0.05.

## Discussion

This study is the first that investigates suPAR in asthma and asthmatic pregnancy. According to our results, although treated asthma generally is not associated with altered suPAR levels (compared to healthy non-pregnant control data), in asthmatic (non-pregnant) patients high suPAR indicated impaired lung function. Furthermore, a cut-off value of suPAR was detected which proved to be suitable to discriminate with high sensitivity between ANP patients with controlled or uncontrolled disease. On the other hand, a suPAR was consistently lower in pregnant subjects regardless of asthma (compared to respective healthy and asthmatic non-pregnant values).

Many studies have shown that increased levels of suPAR are related to worse prognosis in diseases with active immune response such as rheumathoid arthritis [27], infections [25], and pathological pregnancies [30]. Asthma is also associated with systemic inflammation related to lung function and clinical symptoms [1]. Pregnancy on the other hand is characterized by immune tolerance resulting in attenuation of immunological responses [13]. Considerable amount of data support that impaired maternal tolerance is responsible for adverse neonatal outcomes in gestations complicated with uncontrolled asthma. In our earlier study, fetal growth restriction was related to active, asthma-associated maternal inflammatory responses in symptomatic asthmatic pregnancy [34]. Therefore, it may be speculated that fetal well-being and optimal growth might depend on sufficient regulation of immune responses during pregnancy and also that decrease in suPAR level in pregnant groups in this study (regardless of the presence of mostly controlled asthma) was the sign of the immune tolerance caused by pregnancy.

Although Yokoyama and coworkers have reported that patients with stable allergic and non-allergic asthma exhibited an increase in circulating IL-6 [5], our results show comparable IL-6 levels in all study groups. This apparent contradiction between our data and previous results may be based on different definition of stable asthma and lack of inhaled steroid treatment in the latter but not in our study. Furthermore, one should note that the most marked alteration of IL-6 levels was detected during asthmatic attack in the studies of Yokoyama.

It is known from recent studies that CRP is elevated in asthma and that high CRP is associated with respiratory impairment or bronchial hyperresponsiveness [2], [35], [36]. Other studies showed that CRP levels are only elevated in steroid naïve asthmatic patients, but not in those taking inhaled corticosteroids (ICS) regularly [37]. In our experiments CRP was comparable among the four study groups, except for a limited increase measured in asthmatic pregnant compared to healthy non-pregnant but not healthy pregnant group. This is in agreement with previous findings and can be explained by the fact that most of the asthmatic patients examined in this study were taking ICS. On the other hand, the small elevation of CRP in asthmatic pregnant women could be explained with non-adherence to steroid treatment as pregnant asthmatics are known to be less compliant to take the prescribed ICS regularly [**10**]. Thus, a limitation of our study is the lack of known steroid-naïve patients. A further confounding factor may be the age difference of patients as asthmatic non-pregnant patients were older than AP women; however it must be noted that suPAR levels are not expected to be influenced by this slight difference of age [38].

Little is known about inflammatory mechanisms inducing increased production of mucus, causing mucosal edema or hypertrophy of the smooth muscles in the bronchi, but all these changes increase airway resistance and severity of asthma [39], [40]. Whether suPAR and IL-6 play a role in regulation of the above processes needs further investigation. To test their potential contribution, PEF, Raw and ACT values were correlated with suPAR and IL-6 levels in asthmatic groups and a positive correlation was found both between suPAR and Raw and between IL-6 and Raw, suggesting a possible role of these inflammatory molecules in the development of asthmatic airway narrowing and increased airway resistance.

The potential value of CRP, suPAR and IL-6 in the determination of asthma control was analyzed using ROC analysis. Since the current asthma guideline suggests PEF>80% predicted and ACT total score>20 as the main determinants of well-controlled asthma, ROC analyses of asthmatic patients’ data were performed in subgroups of patients with PEF above and below 80% and ACT total score above and below 20. ROC analysis of suPAR values based on PEF yielded a cut-off point of 4.04 ng/ml, with an AUC of 0.75. A slightly better performance of suPAR was measured when patients were discriminated on the basis of ACT, where AUC was 0.8 at a cut-off value of 4.04 ng/ml. This could be explained by the fact that ACT measures diversified symptoms (daytime symptom frequency, rescue therapy, sleep frequency and activity limitations; [41]) and, unlike PEF, did not provide information exclusively about the ability to breath. Of note, the diagnostic performance of suPAR was much lower in asthmatic pregnancy than in non-pregnant asthmatics. Furthermore, the cut-off values have a satisfactory sensitivity coupled with limited specificity, indicating that the sole usage of suPAR as a marker of asthma control might overestimate uncontrolled patients.

In summary, suPAR is a promising biomarker of asthma control in asthmatic non-pregnant patients, since it correlates with airway resistance and has good sensitivity in the detection of impaired asthma control. However, future studies are needed to demonstrate whether the suggested diagnostic value of suPAR would translate into clinical practice. A pregnancy specific decrease can be detected in circulating suPAR levels both in healthy and asthmatic pregnant women. Theoretically, this may be the result of immune tolerance in pregnancy and the attenuation of systemic inflammatory responses.

## References

[1] Global Initiative for Asthma. Available: http://www.ginasthma.org. Accessed 17 September 2012.

[2] Kony S, Zureik M, Driss F, Neukirch C, Leynaert B, et al (2004) Association of bronchial hyperresponsiveness and lung function with C-reactive protein (CRP): a population based study. Thorax 59: 892–896. 15454657 10.1136/thx.2003.015768 PMC1746828

[3] Allam MH, Said AF, El Samie Omran AA, Abd El-Reheim DM, Kasem AH (2009) High sensitivity C-reactive protein: its correlation with sputum cell counts in bronchial asthma. Respir Med 103: 1878–1884. 19836939 10.1016/j.rmed.2009.06.020

[4] Rose-John S, Scheller J, Elson G, Jones SA (2006) Interleukin-6 biology is coordinated by membrane-bound and soluble receptors: role in inflammation and cancer. J Leukoc Biol 80: 227–236. 16707558 10.1189/jlb.1105674

[5] Yokoyama A, Kohno N, Fujino S, Hamada H, Inoue Y, et al (1995) Circulating interleukin-6 levels in patients with bronchial asthma. Am J Respir Crit Care Med 151: 1354–1358. 7735584 10.1164/ajrccm.151.5.7735584

[6] Higashimoto Y, Yamagata Y, Taya S, Iwata T, Okada M, et al (2008) Systemic inflammation in chronic obstructive pulmonary disease and asthma: similarities and differences. Respirology 13: 128–133. 18197923 10.1111/j.1440-1843.2007.01170.x

[7] Kwon H, Belanger K, Bracken MB (2003) Asthma prevalence among pregnant and childbearing-aged women in the United States: estimates from national health surveys. Ann Epidemiol 13: 317–324. 12821270 10.1016/s1047-2797(03)00008-5

[8] Demissie K, Breckenridge MB, Rhoads GG (1998) Infant and maternal outcomes in the pregnancies of asthmatic women. Am J Respir Crit Care Med 158: 1091–1095. 9769265 10.1164/ajrccm.158.4.9802053

[9] Breton MC, Beauchesne MF, Lemière C, Rey E, Forget A, et al (2009) Risk of perinatal mortality associated with asthma during pregnancy. Thorax 64: 101–106. 19008298 10.1136/thx.2008.102970

[10] Murphy VE, Clifton VL, Gibson PG (2006) Asthma exacerbations during pregnancy: incidence and association with adverse pregnancy outcomes. Thorax 61: 169–176. 16443708 10.1136/thx.2005.049718 PMC2104591

[11] Toldi G, Molvarec A, Stenczer B, Müller V, Eszes N, et al (2011) Peripheral T(h)1/T(h)2/T(h)17/regulatory T-cell balance in asthmatic pregnancy. Int Immunol 23: 669–677. 21937455 10.1093/intimm/dxr074

[12] Tamási L, Bohács A, Tamási V, Stenczer B, Prohászka Z, et al (2010) Increased circulating heat shock protein 70 levels in pregnant asthmatics. Cell Stress Chaperones 15: 295–300. 19777374 10.1007/s12192-009-0143-8 PMC2866990

[13] Tamási L, Horváth I, Bohács A, Müller V, Losonczy G, et al (2011) Asthma in pregnancy – Immunological changes and clinical management. Respir Med 105: 159–164. 21145223 10.1016/j.rmed.2010.11.006

[14] Murphy VE, Gibson PG (2011) Asthma in pregnancy. Clin Chest Med 32: 93–110. 21277452 10.1016/j.ccm.2010.10.001

[15] Schatz M, Dombrowski MP, Wise R, Lai Y, Landon M, et al (2010) The relationship of asthma-specific quality of life during pregnancy to subsequent asthma and perinatal morbidity. J Asthma 47: 46–50. 20100020 10.3109/02770900903483758 PMC3249656

[16] Danø K, Behrendt N, Brünner N, Ellis V, Ploug M, et al (1994) The urokinase receptor. Protein structure and role in plasminogen activation and cancer invasion. Fibrinolysis 8: 189–203.

[17] Behrendt N, Stephens RW (1998) The urokinase receptor. Fibrinolysis and Proteolysis 12: 191–204.

[18] Stephens RW, Pedersen AN, Nielsen HJ, Hamers MJ, Høyer-Hansen G, et al (1997) ELISA determination of soluble urokinase receptor in blood from healthy donors and cancer patients. Clin Chem 43: 1876–1884. 9342006

[19] Rønne E, Pappot H, Grøndahl-Hansen J, Høyer-Hansen G, Plesner T, et al (1995) The receptor for urokinase plasminogen activator is present in plasma from healthy donors and elevated in patients with paroxysmal nocturnal haemoglobinuria. Br J Haematol 89: 576–581. 7734357 10.1111/j.1365-2141.1995.tb08366.x

[20] Sier CF, Sidenius N, Mariani A, Aletti G, Agape V, et al (1999) Presence of urokinase-type plasminogen activator receptor in urine of cancer patients and its possible clinical relevance. Lab Invest 79: 717–722. 10378514

[21] Riisbro R, Christensen IJ, Høgdall C, Brünner N, Høgdall E (2001) Soluble urokinase plasminogen activator receptor measurements: influence of sample handling. Int J Biol Markers 16: 233–239. 11820717 10.1177/172460080101600402

[22] Ostrowski SR, Katzenstein TL, Piironen T, Gerstoft J, Pedersen BK, et al (2004) Soluble urokinase receptor levels in plasma during 5 years of highly active antiretroviral therapy in HIV-1-infected patients. J Acquir Immune Defic Syndr 35: 337–342. 15097149 10.1097/00126334-200404010-00002

[23] Ostrowski SR, Ullum H, Goka BQ, Høyer-Hansen G, Obeng-Adjei G, et al (2005) Plasma concentrations of soluble urokinase-type plasminogen activator receptor are increased in patients with malaria and are associated with a poor clinical or a fatal outcome. J Infect Dis 191: 1331–1341. 15776381 10.1086/428854

[24] Ostrowski SR, Ravn P, Hoyer-Hansen G, Ullum H, Andersen AB (2006) Elevated levels of soluble urokinase receptor in serum from mycobacteria infected patients: still looking for a marker of treatment efficacy. Scand J Infect Dis 38: 1028–1032. 17148072 10.1080/00365540600868305

[25] Ostergaard C, Benfield T, Lundgren JD, Eugen-Olsen J (2004) Soluble urokinase receptor is elevated in cerebrospinal fluid from patients with purulent meningitis and is associated with fatal outcome. Scand J Infect Dis 36: 14–19. 15000553 10.1080/00365540310017366

[26] Balabanov R, Lisak D, Beaumont T, Lisak RP, Dore-Duffy P (2001) Expression of urokinase plasminogen activator receptor on monocytes from patients with relapsing-remitting multiple sclerosis: effect of glatiramer acetate (copolymer 1). Clin Diagn Lab Immunol 8: 1196–1203. 11687463 10.1128/CDLI.8.6.1196-1203.2001 PMC96249

[27] Toldi G, Bekó G, Kádár G, Mácsai E, Kovács L, et al (2012) Soluble urokinase plasminogen activator receptor (suPAR) in the assessment of inflammatory activity of rheumatoid arthritis patients in remission. Clin Chem Lab Med 0: 1–6 doi:10.1515/cclm-2012-0221. 10.1515/cclm-2012-0221 22718576

[28] Sier CF, Stephens R, Bizik J, Mariani A, Bassan M, et al (1998) The level of urokinase-type plasminogen activator receptor is increased in serum of ovarian cancer patients. Cancer Res 58: 1843–1849. 9581823

[29] Odden N, Henriksen T, Mørkrid L (2012) Serum soluble urokinase plasminogen activator receptor (suPAR) in early pregnancy prior to clinical onset of preeclampsia. Acta Obstet Gynecol Scand 91: 1226–1232 doi: 10.1111/j.1600-0412.2012.01504.x. 22774918 10.1111/j.1600-0412.2012.01504.x

[30] Toldi G, Bíró E, Szalay B, Stenczer B, Molvarec A, et al (2011) Soluble urokinase Plasminogen Activator Receptor (suPAR) levels in healthy pregnancy and preeclampsia. Clin Chem Lab Med 49: 1873–1876. 21722073 10.1515/CCLM.2011.656

[31] Backes Y, van der Sluijs KF, Mackie DP, Tacke F, Koch A, et al (2012) Usefulness of suPAR as a biological marker in patients with systemic inflammation or infection: a systematic review. Intensive Care Med 38: 1418–1428. 22706919 10.1007/s00134-012-2613-1 PMC3423568

[32] Eugen-Olsen J, Gustafson P, Sidenius N, Fischer TK, Parner J, et al (2002) The serum level of soluble urokinase receptor is elevated in tuberculosis patients and predicts mortality during treatment: a community study from Guinea-Bissau. Int J Tuberc Lung Dis 6: 686–692. 12150480

[33] Miller MR, Hankinson J, Brusasco V, Burgos F, Casaburi R, et al (2005) Standardisation of spirometry. Eur Respir J 26: 319–338. 16055882 10.1183/09031936.05.00034805

[34] Tamási L, Bohács A, Pállinger E, Falus A, Rigó J Jr, et al (2005) Increased interferon-gamma- and interleukin-4-synthesizing subsets of circulating T lymphocytes in pregnant asthmatics. Clin Exp Allergy 35: 1197–1203. 16164448 10.1111/j.1365-2222.2005.02322.x

[35] Rifai N, Ridker PM (2003) Population distributions of C-reactive protein in apparently healthy men and women in the United States: implication for clinical interpretation Clin Chem. 49: 666–669. 10.1373/49.4.666 12651826

[36] Ford ES (2003) Asthma, body mass index, and C-reactive protein among US adults. J Asthma 40: 733–739. 14626329 10.1081/jas-120023497

[37] Takemura M, Matsumoto H, Niimi A, Ueda T, Matsuoka H, et al (2006) High sensitivity C-reactive protein in asthma. Eur Respir J 27: 908–912. 16707391 10.1183/09031936.06.00114405

[38] Thunø M, Macho B, Eugen-Olsen J (2009) suPAR: the molecular crystal ball. Dis Markers 27: 157–172. 19893210 10.3233/DMA-2009-0657 PMC3835059

[39] Light RW (2005) Mechanics of respiration. In: Ronald BG, Light RW, Matthay MA, Matthay RA. Chest Medicine: Essentials of Pulmonary and Critical Care Medicine. New York: Lippincott Williams & Wilkins. pp. 24-38.

[40] Neveu WA, Allard JL, Raymond DM, Bourassa LM, Burns SM, et al (2010) Elevation of IL-6 in the allergic asthmatic airway is independent of inflammation but associates with loss of central airway function. Respir Res 11: 28. 20205953 10.1186/1465-9921-11-28 PMC2842243

[41] Cloutier MM, Schatz M, Castro M, Clark N, Kelly HW, et al. (2012) Asthma outcomes: composite scores of asthma control. J Allergy Clin Immunol 129 (3 Suppl): S24-33. 10.1016/j.jaci.2011.12.980 PMC4269334 22386507

